# Fibertools: fast and accurate DNA-m6A calling using single-molecule long-read sequencing

**DOI:** 10.1101/2023.04.20.537673

**Published:** 2023-07-06

**Authors:** Anupama Jha, Stephanie C. Bohaczuk, Yizi Mao, Jane Ranchalis, Benjamin J. Mallory, Alan T. Min, Morgan O. Hamm, Elliott Swanson, Connor Finkbeiner, Tony Li, Dale Whittington, William Stafford Noble, Andrew B. Stergachis, Mitchell R. Vollger

**Affiliations:** 1. Department of Genome Sciences, University of Washington, Seattle, WA, USA; 2. Division of Medical Genetics, University of Washington School of Medicine, Seattle, WA, USA; 3. Department of Statistics, University of Washington, Seattle, WA, USA; 4. Department of Medical Chemistry, University of Washington, Seattle, WA, USA; 5. Paul G. Allen School of Computer Science and Engineering, University of Washington, Seattle, WA, USA; 6. Brotman Baty Institute for Precision Medicine, Seattle, WA USA

## Abstract

Single-molecule chromatin fiber sequencing is based on the single-nucleotide resolution identification of DNA *N*^6^-methyladenine (m6A) along individual sequencing reads. We present fibertools, a semi-supervised convolutional neural network that permits the fast and accurate identification of both endogenous and exogenous m6A-marked bases using single-molecule long-read sequencing. Fibertools enables highly accurate (>90% precision and recall) m6A identification along multi-kilobase DNA molecules with a ~1,000-fold improvement in speed and the capacity to generalize to new sequencing chemistries.

## Introduction

**Figure 1.**
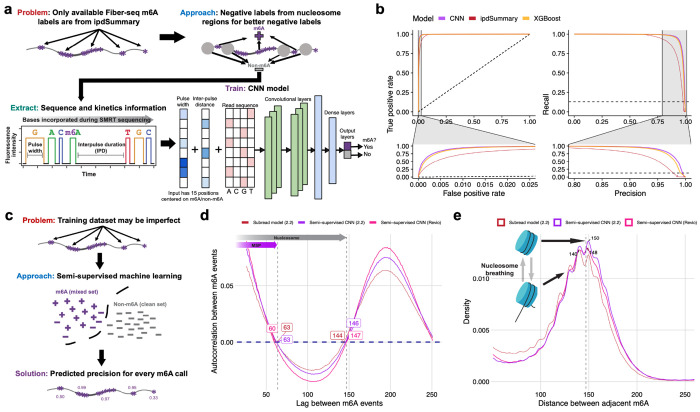
Accurate identification of m6A with machine learning (ML) and refinement with semi-supervised ML. **a)** Methodology for generating training data and identifying m6A modifications using PacBio HiFi (see Methods for details). **b)** Receiver operating characteristic and precision-recall curves for the CNN (purple), XGBoost (orange), and ipdSummary (red) models. Dashed lines indicate the performance of a random classifier. **c)** Methodology for semi-supervised machine learning (see Methods for details). **d)** Autocorrelation between m6A calls made by the subread (red), semi-supervised (purple), and Revio (pink) models. **e)** Density of the distance between adjacent m6A on the same chromatin fiber (10,000 reads) for the same datasets and models as in d.

Highly accurate long-read single-molecule sequencing has revolutionized the comprehensive assembly of phased genetic architectures (Wenger et al. 2019; Vollger et al. 2020; Nurk et al. 2022). In addition, long-read single-molecule sequencing has permitted the direct identification of modified DNA bases such as m6A and 5-methylcytosine (5mC) (Marks et al. 2012; Clark et al. 2012; Murray et al. 2012; Loman et al. 2015; [CSL STYLE ERROR: reference with no printed form.]) enabling single-molecule chromatin fiber sequencing (Stergachis et al. 2020; Lee et al. 2020; Abdulhay et al. 2020; Shipony et al. 2020). Specifically, single-molecule chromatin fiber sequencing leverages non-specific methyltransferases to selectively stencil chromatin protein occupancy patterns directly onto their underlying DNA molecules in the form of modified bases. Modified bases along individual DNA molecules are then directly identified using PCR-free single-molecule sequencing. For example, during single-molecule, real-time (SMRT) sequencing, the identity of each base is determined based on the fluorophore-labeled nucleotide that is incorporated as the polymerase replicates the base. In contrast, the modification status of each base is determined based on signature changes in polymerase kinetics at and surrounding that base as it is replicated by the polymerase, such as elongation of the interpulse duration (IPD) due to polymerase pausing at modified bases (Flusberg et al. 2010) (Fig. 1a and Fig S1). Recently developed tools leverage these polymerase kinetic parameters to identify 5mC within specific sequence contexts (Tse et al. 2021; [CSL STYLE ERROR: reference with no printed form.]), genomic positions with consistent m6A signal across multiple sequencing reads (Marks et al. 2012; Clark et al. 2012; Murray et al. 2012), as well as total adenine methylation levels along very short sequencing reads (Kong et al. 2022). However, identifying m6A-modified bases at single-molecule and single-nucleotide resolution has proven more computationally challenging, with current computational tools, such as ipdSummary and SAMOSA-ChAAT requiring excessive compute and storage resources (Fig. S2) and lacking the ability to accurately call methylation along multi-kilobase reads, or operate with modern SMRT sequencing chemistry that does not include subread files (Fig. S3) (Abdulhay et al. 2021; Dubocanin et al. 2022). These limitations have throttled the ability of single-molecule chromatin fiber sequencing to scale to mapping chromatin across large genomes, such as the human genome.

## Results

Building an accurate tool for m6A identification requires a training dataset of multi-kilobase reads with both methylated and unmethylated adenines across diverse sequence contexts (i.e., all possible 7-mers containing a central adenine) and methylation density contexts (i.e., m6A in isolation or clustered together). Because creating such a dataset is not achievable using synthetic DNA or fully methylated and unmethylated samples, we sought to determine whether we could use DNA from single-molecule chromatin fiber sequencing reactions (i.e., Fiber-seq) as the basis for training. Specifically, Fiber-seq uses non-specific m6A-MTases to selectively mark sites of protein occupancy along individual DNA molecules via m6A-marked bases. Because protein occupancy is highly heterogeneous across chromatinized DNA (Fig. S4), each DNA molecule contains methylated adenines within diverse sequence and methylation density contexts (Fig. S5). Furthermore, we can employ chromatin features, such as nucleosome occupancy, to bolster training and validation, making Fiber-seq well suited for training a general-purpose m6A caller given labeled data.

To generate initial positive and negative labels, we adapted an existing m6A caller (ipdSummary) that requires subreads for each sequencing read, which are no longer saved with new sequencing chemistries owing to their excessive size (~1 TB) (Fig. S2). IpdSummary normalizes IPDs for their sequence context to create IPD ratios (ipdRatios), and then we use a Gaussian mixture model to identify adenines with ipdRatios that significantly deviate from the expected distribution of unmethylated adenines (i.e., m6A-modified bases) (Dubocanin et al. 2022). These calls are then used to identify m6A-modified bases (positive labels). Negative labels are drawn from regions with extended stretches devoid of m6A corresponding to nucleosome-occluded regions (Fig 1a, Methods), because the false negative rate within nucleosome footprints should be substantially lower than the false negative rate of each individual call.

Using this training dataset, we tested whether we could identify m6A-modified bases using summary kinetic data from each sequencing read routinely produced with SMRT sequencing, as this would bypass the need for subread kinetic information (Fig. 1a). Using this dataset, we independently trained two machine-learning models, XGBoost (Chen and Guestrin 2016) and a convolutional neural network (CNN), and we evaluated their performance on a held-out dataset from a separate sequencing experiment (Fig 1a,b, and Methods). Both models outperformed ipdSummary in terms of AUROC and AUPR (Table S1), achieving an average precision over 97%. Overall, the CNN model had the best performance, and we used it as the basis for subsequent improvements and validation.

Given the limitation of deriving training data from ipdSummary, we set out to refine our CNN model with a semi-supervised training regime (Methods), “fibertools”, which allows for the possibility that the positive labels from ipdSummary are inaccurate (Fig. 1c) (Käll et al. 2007; Fondrie and Noble 2021). Direct use of labels from training data in semi-supervised machine learning does not provide an accurate assessment of performance, so we established a series of biological validations to test the performance of the semi-supervised training.

First, we assessed the accuracy of fibertools for identifying nucleosome footprints along Fiber-seq data. Using single-molecule m6A calls with a predicted precision of >95% (Methods), we performed an autocorrelation analysis. Compared to the fully supervised model, fibertools more accurately recapitulated the exact length of nucleosomes (147 bp) (Fig. 1d) (Luger et al. 1997) and showed an overall higher amplitude autocorrelation, consistent with higher quality identification of m6A. In addition, comparing the distance between adjacent m6A methylation marks in human Fiber-seq data demonstrated clear oscillatory patterns suggestive of nucleosome breathing (Polach and Widom 1995; Anderson and Widom 2000; Hall et al. 2009), further indicative of high-quality m6A identification (Fig. 1e).

**Figure 2.**
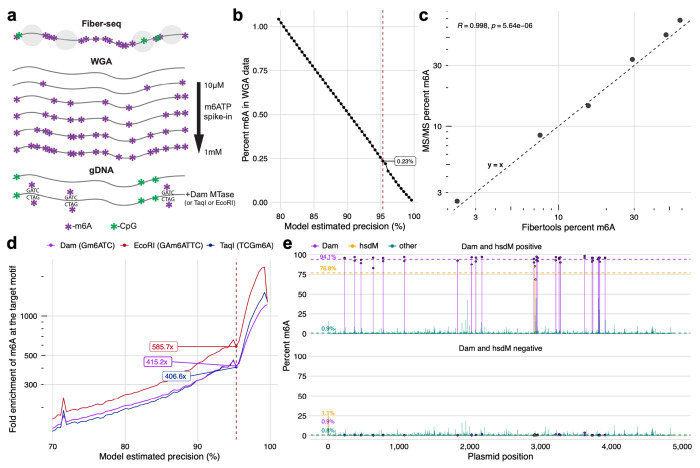
Biological validation of the semi-supervised m6A caller. **a)** Description of biological samples used for validation of fibertools. **b)** Percent of methylated adenines called by fibertools relative to all adenines in a whole-genome amplified (WGA) negative control as a function of the estimated precision reported by fibertools. This serves as an estimate of the false positive rate. The red line marks the default threshold used by fibertools. **c)** Percent m6A as determined by UHPLC-MS/MS (y) and fibertools (x) at the default precision level for WGA samples with varying levels of m6ATP spiked-in. The text (upper left) indicates the value of the Pearson’s correlation coefficient and the P value from a two-sided t-test without adjustment for multiple comparisons. **d)** Enrichment of m6A calls within targeted motifs of three motif-specific methyltransferases [Dam (blue), EcoRI (orange), and TaqI (green)] as a function of fibertools estimated precision, **e)** Methylation percent at recognition sites for *Dam* (purple), *HsdM* (orange), and other sites (green) among all sequencing reads of a plasmid grown in a dam^+^/hsdM^+^ bacterial strain (top) compared to a dam^−^/hsdM^−^ negative control (bottom). Dotted lines show the average across each category.

Second, we evaluated the false positive rate (FPR) of fibertools using whole-genome amplified (WGA) DNA that lacked m6A (Fig. 2a). Our findings indicated an FPR of 0.23% at the model-predicted precision level of >95% (Fig. 2b and Fig. S6). Notably, given this FPR, this approach is not suited for identification of non-specific genomic m6A events within species with low-level endogenous m6A (Kong et al. 2022; Debo et al. 2023).

Third, we evaluated the ability of fibertools to accurately quantify the total amount of m6A within a sample. Specifically, we spiked varying levels of m6ATP into a WGA reaction (Fig. 2a) and employed ultra-high-performance liquid chromatography tandem mass spectrometry (UHPLC–MS/MS) to determine the percentage of methylated adenines with respect to all adenines (Methods). We then sequenced these samples, applied fibertools, and found a strong correlation (Pearson=0.998, p-value=5.2e-6) between our method and mass spectrometry (Fig. 2c).

Fourth, we evaluated the precision of fibertools for identifying m6A within various motif contexts using genomic DNA treated with motif-specific methyltransferases. We found that m6A calls were enriched by 415-fold, 586-fold, and 407-fold in motifs specific to Dam, EcoRI, and TaqI, respectively (Fig. 2d), consistent with precise m6A calls.

Fifth, we evaluated the accuracy of fibertools for identifying endogenously m6A-modified bases within bacteria. Notably, DNA isolated from bacteria expressing both the Dam and HsdM methyltransferases exhibited m6A at 94.1% of the target Dam sites for these methyltransferases, indicating a false negative rate of less than 6% in this sequence context (Fig. 2e). In contrast, DNA from bacteria lacking these methyltransferases exhibited m6A at <1% of adenines, consistent with our prior FPR estimate. Collectively, these five biological validations provide strong evidence that fibertools is highly accurate and specific in identifying m6A events using PacBio HiFi data.

**Figure 3.**
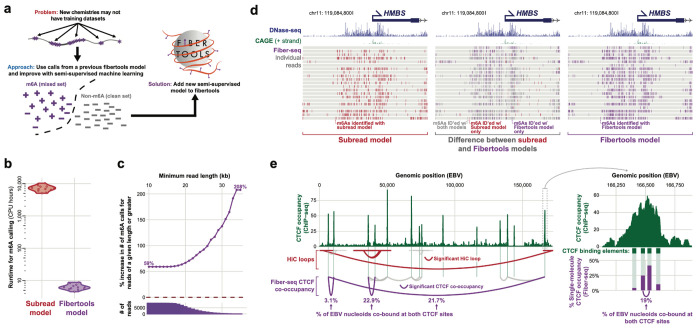
Increased speed and throughput via fibertools. **a)** Methodology for generalizing fibertools for new chemistries using semi-supervised ML and models from existing chemistries. **b)** CPU hours used by fibertools (purple) and subread-based GMM model (red) for individual SMRT cells. Fibertools was run with GPU acceleration (NVIDIA A40), which is unavailable for the GMM model. **c)** Percent increase in fibertools m6A calls over the GMM model as a function of the minimum read length of the underlying sequencing data. The histogram below shows how many reads were used to calculate each percent increase. **d)** Visualization of m6A calls in the *HMBS* locus that are unique to fibertools (purple), unique to the subread GMM model (red), or shared by both (gray). Reads are sorted by the number of CCS passes. DNase-seq signal is shown above **e)** *left* CTCF ChIP-seq (green), HiC loops (red), and CTCF site co-occupancy by Fiber-seq (purple) along the EBV genome. *right* Zoom-in of the indicated CTCF peak, which contains four CTCF binding elements. Single-molecule occupancy and co-occupancy from Fiber-seq are shown below.

Given the performance of fibertools in accurately accounting for putative false positives in the training data, we next tested whether this approach would enable us to readily adapt our model to new sequencing chemistries, which often contain updated polymerases that may differ in their kinetic values (Fig. 3a). As proof of principle, we used the model from the PacBio Sequel II 2.2 chemistry as the initialization point for training a semi-supervised model for the PacBio Revio chemistry. Using the biological validations above, we show that the semi-supervised Revio model is highly accurate in identifying m6A events (Fig. 1d,e).

To improve the functionality of fibertools, we optimize it using a compiled language, provide it as a single binary accessible through bioconda (package “fibertools-rs”), and integrate m6A calls directly into the BAM format using the MM and ML tags (Fig. S7). Fibertools can process individual SMRT cells in 5-8 CPU hours, a >1,000-fold increase in speed compared to the previous pipeline when using GPU acceleration (>150-fold increase without GPU) (Fig. 3b and Table S2).

Finally, in addition to speed, fibertools also substantially reduces the amount of false-negative methylation calls (Fig 3c,d), an improvement primarily driven by enabling m6A calling along multi-kilobase reads with fewer subread passes—a limitation of prior m6A calling tools (Fig. S8). To demonstrate the utility of m6A-calls along multi-kilobase fibers, we sought to resolve the role of CTCF co-occupancy in guiding higher-order chromatin architectures along the ~175 kbp Epstein-Barr virus (EBV) genome from GM12878 cells. CTCF elements within the EBV OriP and LMP loci are known to form a cohesin-dependent loop important for maintaining viral latent cycle gene expression (Arvey et al. 2012; Morgan et al. 2022). We find that CTCF occupancy within the *LMP* locus is mainly mediated by two adjacent CTCF elements that are preferentially co-bound by CTCF along the same EBV nucleoid (Fig. 3e). Furthermore, EBV nucleoids bound by CTCF within the *LMP* locus are also preferentially co-bound by a single CTCF element within the EBV OriP locus, which is located ~12 kbp away (Fig. 3e). However, only 21.7% of EBV nucleoids are co-bound by CTCF at both the OriP and *LMP* loci, setting an upper limit on the proportion of EBV nucleoids that are structured by CTCF-mediated three-dimensional looping between these two loci. These findings suggest that dynamic CTCF looping, which has been observed along the nuclear genome (Gabriele et al. 2022), is mediated by CTCF co-occupancy across interacting sites in EBV nucleoids.

## Discussion

In summary, fibertools enables highly accurate single-molecule DNA-m6A identification along 20+ kilobase DNA fibers, with a 1,000-fold improvement in speed and a 10-fold reduction in data storage usage. Furthermore, unlike all existing tools for m6A identification, fibertools works directly with summary kinetic information produced during SMRT sequencing, obviating the need for subread files that are often multiple terabytes in size and are no longer produced with modern SMRT sequencing instruments (Abdulhay et al. 2021; Dubocanin et al. 2022). These advances permit deep-coverage, single-molecule chromatin fiber sequencing to scale to large genomes, enabling the identification of single-molecule protein occupancy events, as well as the co-dependency between protein occupancy along individual chromatin fibers. Finally, the training structure for fibertools enables it to readily adapt to new sequencing chemistries as they emerge.

## Methods

### Initial m6A calling with ipdSummary followed by filtering with a GMM

To initially identify m6A, we use a previously published protocol (Dubocanin et al. 2022) with the following details and modifications. Raw PacBio subread BAM files were converted into CCS (circular consensus sequence) reads using pbccs (v6.0.0) (https://ccs.how/) with average kinetics information included. Then subreads were aligned to their respective CCS reads using actc (https://github.com/PacificBiosciences/actc). The resulting alignments were passed to ipdSummary (v3.0) (https://github.com/PacificBiosciences/kineticsTools/) to identify positions with m6A modifications with the CCS-specific extracted genomic sequence as the reference and these additional flags: --pvalue 0.001 --identify m6A. For each read, we then trained a two-component Gaussian mixture model (GMM) from the ipdRatios generated by ipdSummary for all adenine bases within the read (push_m6a_to_bam.py). Adenine bases were then classified as m6A modified if the probability that the ipdRatio came from the larger distribution was greater than or equal to 0.99999999. All code to repeat these steps is made available as a single snakemake (Köster and Rahmann 2012) pipeline on GitHub (https://github.com/StergachisLab/fiberseq-smk).

### Hidden Markov model nucleosome calling

To identify nucleosomes initially, we use a previously published protocol (Dubocanin et al. 2022) with the following details and modifications. We used m6A calls to train a two-state (nucleosome, non-nucleosome) Hidden Markov model (HMM) with a standardized post-hoc correction. The input data for the HMM skipped all G/C bases and considered only the A/T sequence so that GC-rich regions without adenine would not falsely be called nucleosomes. The HMM was then trained using the Baum-Welch algorithm (Baum et al. 1970) using a subsample of 5,000 CCS reads per SMRT cell, and the trained model was subsequently applied across all fibers from that SMRT cell using the Python package pomegranate (Schreiber 2017). In tandem with the HMM delineation of nucleosomes, we also applied a simplified approach that looked for unmethylated stretches larger than 85 bp in length (irrespective of A/T content). We used these ‘simple’ calls to refine our HMM nucleosome calls by splitting large HMM nucleosomes that contained multiple ‘simple’ calls. We also refined the terminal boundaries of each nucleosome by bookending it to the nearest m6A call within 10 bp, if present. Nucleosome calling is available as part of a snakemake pipeline (https://github.com/StergachisLab/fiberseq-smk) and encodes the resulting calls in the custom BAM flags ns (nucleosome start) and nl (nucleosome length), which can be extracted using fibertools-rs.

### Generating positive and negative labels for training and validation data

To generate positive labels, we used the previously existing calls made using the GMM-filtered calls from ipdSummary in our snakemake pipeline. Negative labels were drawn from nucleosome regions as defined by the HMM since we had increased confidence that these regions should be inaccessible due to the presence of a nucleosome. For each SMRT cell, we selected ~350,000 CCS reads and randomly sampled 5% of available positive and negative m6A positions within each read to reduce the number of adjacent and, therefore, non-independent calls in our training data. This resulted in a dataset of ~100 million negative labels and ~8 million positive labels for each SMRT cell. We note that by selecting negative labels from within nucleosomes, we allow for the possibility for our models to outperform ipdSummary and the GMM correction even though we draw positive labels from these tools. For each label in our dataset, we included a 15 bp window centered around the adenine encoding the sequencing information with one-hot encoding and the CCS kinetics information across the same 15 bases for pulse width and interpulse duration resulting in a 6 X 15 matrix for each labeled position. Code to generate training data from CCS reads is available on GitHub (https://github.com/mrvollger/m6A-calling).

### Training, validation, and testing datasets for machine learning

For the 2.2 PacBio sequencing chemistry, we established three separate datasets, each from a different sequencing run, for developing our models. The three datasets were used for training, validation, and a completely held-out testing dataset for determining final accuracies. Data for v2.2 chemistry was generated from previously described samples (K562, CHM1) treated with 200 units of Hia5 for 10 minutes at 25°C (Dubocanin et al. 2022). Data for v3.2 chemistry was generated from a K562 Fiber-seq sample (described below), and Revio data was provided by PacBio.

### Architectures and training of machine-learning models (XGBoost, CNN, and semi-supervised CNN)

We trained an XGBoost model with a binary logistic objective function using our training and validation datasets. We selected the following hyperparameters using threefold cross-validation; learning rate of 1 (gamma), maximum tree depth of 8, minimum child weight of 100, and 150 estimators. The code to repeat this training is available on GitHub (https://github.com/fiberseq/train-m6A-calling).

### Convolutional neural network

We trained a CNN to predict m6A in Fiber-seq HiFi reads. Input to the CNN model is the 6x15 matrix described above. The model has three convolutional layers with 30, 10 and 5 filters of size 5, 5, and 3, respectively. A dense layer of size 25 x 5 and an output layer of size 5 x 2 follow the convolutional layers. All internal layers have ReLU activation, and the output layer has softmax activation. The output layer has two classes, one for m6A and one for unmethylated adenines. Each class generates scores between 0 and 1 for each input matrix, where scores close to 1 denote high confidence that the input belongs to that class. For example, unmethylated adenines score close to 0 for the m6A class, and methylated adenines score close to 1. The CNN optimizes a binary cross-entropy loss function using the Adam optimizer (Kingma and Ba 2014). We trained the model iteratively for 30 epochs, where the training data was input in random batches of 32 in each epoch. See Table S3 for the size of training and validation datasets from different Fiber-seq chemistries for supervised training. The code to repeat this training is available on GitHub (https://github.com/fiberseq/train-m6A-calling).

### Semi-supervised convolutional neural network

We derive m6A labels from GMM-filtered ipdRatios from ipdSummary. These labels can contain false positives. The lack of clean m6A labels makes the supervised training approach less suitable since it assumes that accurate labels are available. Therefore, we developed a semi-supervised approach, which assumes that our m6A class has a mixed population of true and false positives and our non-m6A class is a clean set. Our training approach is derived from the Percolator and mokapot proteomics tools for identifying peptides from tandem mass spectrometry data (Käll et al. 2007; Fondrie and Noble 2021). This approach yields a classifier with m6A calls at a target precision. The semi-supervised algorithm is outlined in Supplementary Methods and Supplementary Algorithm 1. First, we split our dataset into training and validation sets stratified by class labels (see Table S3 for the size of training and validation datasets from different Fiber-seq experiment chemistries for semi-supervised training). Then our method proceeds in two phases. In the first phase, we use the IPD score of the central base as a classifier and generate an m6A classification score for all examples in the validation set. The classification score ranks the validation examples, and precision is computed at every score threshold. At the end of this phase, we select a score threshold to achieve the target precision. In this work, we use a target precision of 95%. The second phase is iterative, and each iteration consists of three steps. The first step is selecting a high-confidence m6A training set using the current score threshold. The second step consists of training a CNN model on this training data. In the final step, the validation data is rescored using the trained CNN model from the second step, and a new score threshold is generated at 95% precision with the rescored validation data. In the case of a successful second phase training, the number of positives in the validation data identified at target precision increases with every iteration and plateaus when most m6A examples in the validation data have been identified. We define two conditions for convergence, both of which must be satisfied. First, more than 70% of putative m6A calls from the validation set have been identified. Second, the number of additional m6A calls in a new iteration is less than 1% of the total putative m6A calls. In practice, it took 12, 11, and 3 iterations of phase two training to converge 2.2, 3.2, and Revio chemistry Fiber-seq experiments, respectively (Fig. S9). The code to repeat this training is available on GitHub (https://github.com/fiberseq/train-m6A-calling).

### Encoding and selecting precision levels for m6A calling

Using the approach outlined in the semi-supervised method, we calculated the empirical precision using the validation data for every score output from the CNN model (see Supplementary Methods and Table S4 for details). These precisions are then multiplied by 256 and rounded into an 8-bit integer following the BAM specification for the ML tag (https://samtools.github.io/hts-specs/SAMtags.pdf, (Li et al. 2009). We then chose the first value (244) with a precision greater than 95% (244/256) as the threshold for calling positive m6A events.

### Chemicals and materials used

1 M Tris-HCl (pH8, molecular biology grade ultrapure, ThermoScientific, cat#J22638-K2), 2-deoxyadenosine (A) (>99%, FisherScientific, cat#AAJ6388606), Benzonase (Millipore Sigma, cat#E1014), CpG methyltransferase M.SssI(NEB, cat#M0226), dNTP set (Neta Scientific, cat#GHC-28-4065-51), HMW DNA Extraction kit (Promega, Cat#A2920), M. SssI (NEB, cat#M0226S), N6-methyl-2-dATP (TriLink, cat#N-2025), N6-methyl-2-deoxyadenine (m6A) (>99%, FisherScientific, cat#AAJ64961MD), Nanosep (MWCO 3 kDa, Pall, cat#OD003C33), phosphodiesterase I (Worthington, cat#LS003926), Quick CIP (NEB, cat#M0525S), Ultrapure distilled water (Invitrogen, cat#10977015). Taq methyltransferase (NEB, cat#M0219S), Dam methyltransferase (NEB, cat#M0222S), EcoRI methyltransferase (NEB, M0211S), ProNex^®^ Size-Selective Chemistry (Promega, cat#NG2001), PacBio Elution buffer (PacBio #101-633-500), ZORBAX Eclipse Plus C18 (Agilent, cat#959757-902). All reagents used for mass spectrometer analysis are molecular grade level or above. g-TUBEs, SMRTbell^®^ prep kit 3.0, REPLI-g Mini kit (Cat# 150023).

### Preparation of calcium-competent ER2796 E. coli

A 10mL culture of the ER2796 E. coli strain (obtained from NEB) was grown overnight without antibiotics. 1mL of the overnight culture was added to 99mL of fresh LB media without antibiotics and incubated with shaking at 37°C and 200 rpm for 3-4 hours until OD reached 0.4. The culture was separated into two 50mL falcon tubes and placed on ice for 20 minutes before being pelleted by centrifugation at 4°C and 4000 rpm for 10 minutes. The supernatant was discarded, and the cell pellets were resuspended with 20mL of ice-cold 0.1 M CaCl2 and incubated on ice for 30 minutes. The cells were then pelleted again by centrifugation and the supernatant was discarded. The pellets were then combined by resuspending in 5mL of ice-cold 0.1M CaCl2 with 15% glycerol. Cells were aliquoted in 50ul aliquots, frozen in liquid N2, and stored at −80C.

### Plasmid DNA preparation

A pCS2+ plasmid containing flag-tagged mgfp5 cloned into the EcoRI/XhoI sites (a gift from Lea Starita) was transformed into two strains of competent E. coli, ER2796 (from NEB, see “Preparation of calcium competent ER2796 E. coli”) and NEB 5-alpha (NEB cat. no. C2987I), using a standard heat shock method. 50 μL of the chemically competent cells were thawed on ice and mixed with 50 ng of plasmid DNA. The mixture was placed on ice for 30 minutes before being heat shocked at 42°C for 30 seconds. After heat shock, the cells were immediately placed back on ice for 5 minutes. Following incubation, 950 μL of room temperature SOC media (NEB cat. no. B9020S) was added, and the cells were allowed to outgrow for 1 hour in the absence of selection. 50 μL of the outgrown cells were diluted in 5 mL of selective media and grown overnight with shaking at 37°C and 220 rpm. Specifically, the NEB 5-alpha cells were grown in LB media with 100 μg/mL ampicillin, while the ER2796 cells were grown in LB media with 50 μg/mL kanamycin + 100 μg/mL ampicillin. The following day, plasmid DNA was extracted from the bacterial cells using the Monarch Plasmid Miniprep Kit (NEB cat. no. T1010L) following the manufacturer’s protocol. The elution of plasmid DNA was done with sterile water and the concentration was measured using the Qubit 1X dsDNA HS Assay Kit (Invitrogen cat. no. Q33231).

### K562 cell culture

K562 cells were maintained in suspension in IMDM media supplemented with 10% FBS (HyClone, cat. no.SH30396.03IH25-40) and antibiotic (100 I.U/mL penicillin, 100ug/mL streptomycin, Gibco, cat. no. 15140122) at 37°C and 5% CO2 in T-75 flasks. Cells were split 1:10 every 3-4 days.

### gDNA preparation

Whole-genome DNA was extracted from K562 cells using HMW DNA extraction kit (Promega, Cat# A2920). gDNA was used as a template for all WGA samples. All DNA samples were rehydrated with 10 mM Tris-HCl, pH 8 (ThermoScientific, cat# J22638.K2) or eluted with ultrapure water unless specified.

### WGA preparation

WGA was performed with REPLI-G Mini kit (Cat# 150023) according to the manufacturer’s protocol. 0, 10, 25, 64, 160, 400, or 1000 μM of N6-methyl-2-dATP (TriLink, cat#N-2025) (m6dATP) final concentration was spiked into 50 μL of each WGA to generate samples with 0, 2.56, 8.46, 14.45, 33.42, 52.27, and 67.94% m6A samples, as calculated using mass spec according to an eleven point standard quantified every time with the samples during the same run (see below). Samples were purified with ProNex^®^ Size-Selective Chemistry (Promega, cat#NG2001) by adding a 1:1.3 ratio by volume of sample to magnetic beads. Following incubation for 15 minutes, the sample was placed on a magnetic rack for three minutes, washed twice with 80% ethanol, and eluted in 50 μL of PacBio Elution buffer (PacBio #101-633-500). All m6dATP-spiked samples were purified three times with bead purification, and residual free m6dATP quantified by MS of the 10 and 160 μM m6dATP samples treated with Quick CIP only was near background levels.

### gDNA methylation with sequence-specific methyltransferases

Samples labeled with the sequence-specific adenine methyltransferases EcoRI (GAATTC) (NEB, M0211S), Dam (GATC) (NEB, cat#M0222S), or TaqI (TCGA) (NEB, cat#M0219S) were generated as follows: 400 ng of purified gDNA/purified WGA DNA was treated for one hour with 40U of EcoRI (37°C) (gDNA only), 10U of TaqI (65°C), or 8U of Dam (37°C) in supplied buffer and in the presence of 160 μM SAM. All samples were purified once by ProNex^®^ Size-Selective Chemistry as described above but with a 1:0.8 ratio by volume of sample to magnetic beads.

### Fiber-seq

Non-specific methyltransferase, Hia5, was purified, and the activity was quantified as previously described (Stergachis et al. 2020). Fiber-seq samples were prepared as previously described (Stergachis et al. 2020).

### Library preparation

Samples were sheared in g-TUBEs (Covaris, cat# 520079) for four passes at 3200 RPM for 2-4 minutes in an Eppendorf 5424R centrifuge. Post-shear samples were quantified by Qubit dsDNA high-sensitivity assay (Qubit, cat# Q32851) following the manufacturer’s protocol. Multiplied library preparation was performed using the SMRTbell prep kit 3.0 (PacBio, cat# 102-141-700) and SMRTbell barcoded adapter plate 3.0 (bc2001-bc2019) (PacBio, cat# 102-009-200) according to the manufacturer’s instructions but with the following modifications: after barcoded adapter ligation, samples were incubated at 65°C for 10 minutes to heat-inactivate the ligase. Barcoded samples were pooled and purified with ProNex^®^ Size-Selective Chemistry as described above, with a 1:1 ratio by volume of sample to magnetic beads. Following nuclease treatment, the library was purified first with a 1:3.1 ratio of sample to 35% v/v Ampure PB beads (PacBio, cat# 100-265-900)/PacBio elution buffer. A second purification was performed with a 1:1 ratio of sample to ProNex^®^ Size-Selective Chemistry, as described. The sample was loaded onto a single Sequel II SMRT cell (v3.2 chemistry) and sequenced by the University of Washington PacBio Sequencing Services. The full composition and sample barcode IDs of the multiplexed library are listed in Table S5.

Plasmid DNA was prepared in a separate multiplexed library. Plasmids were linearized with KpnI prior to multiplexed library preparation.

### Quantification of m6A/A by UHPLC-MS/MS

Samples for quantification were treated as previously described with minor modifications (Kong et al. 2022). In brief, 30-50 ng of DNA from each sample was mixed with 0.02 U phosphodiesterase I (Worthington, cat# LS003926), 1 U Benzonase (Millipore Sigma, cat# E1014), and 2 U Quick CIP (NEB, cat#M0525S) in digestion buffer (10 mM Tris, 1 mM MgCl, pH 8 at RT) for 3 hours at 37°C. Single nucleotides were separated from the enzymes by collecting the flow-through of a Nanosep centrifugal filter (MWCO 3 kDa, Pall, cat#OD003C33). The UHPLC-MS/MS analysis of adenosine and m6A was performed on an ACQUITY Premier UPLC System coupled with XEVO-TQ-XS triple quadrupole mass spectrometer. UPLC was performed on a ZORBAX Eclipse Plus C18 column (2.1 × 50 mm I.D., 1.8 μm particle size) (Agilent, cat# 959757-902) using 10-90% linear gradient of solvent B (0.1% acetic acid in 100% methanol) in solvent A (0.1% acetic acid in water) within 4 minutes and a flow rate of 0.3 ml/min. MS/MS analysis was operated in positive ionization mode with 3000 V capillary voltage as well as 150 °C and 1000 L/Hour nitrogen drying gas. A multiple reaction monitoring (MRM) mode was adopted with the following m/z transition: 252.10 →136.09 for dA (collision energy, 14 eV), and 266.2→150.2 for m6A (collision energy, 15 eV). MassLynX was used to quantify the data.

A calibration curve was generated with 11 mixtures containing different ratios of 2-deoxyadenosine (>99%, FisherScientific, cat#AAJ6388606)(A) to N6-methyl-2-deoxyadenine (>99%, FisherScientific, cat# AAJ64961MD)(m6A). A new standard was measured and used for each run. The standard was fit to a third-degree polynomial (equation 1) with y as m6A percentage (%) and x as the quantified MS peak area of m6A over the sum of adenosine and m6A peak area.


[1] y=a1+a2∗x+a3∗x^2+a4∗x^3

## Supplementary Material

Supplement 1

Supplement 2

## Data Availability

All K562 and CHM1 PacBio data was generated as part of a previous study and is available at the following GEO accession: GSE226394. All additional PacBio generated as part of this study is available through the SRA under Bioproject ID: PRJNA956114. Cell lines obtained from the NIGMS Human Genetic Cell Repository at the Coriell Institute for Medical Research include GM12878 and K562. Additional data used in analysis is made available on Zenodo (https://zenodo.org/record/7809229).
